# Bereitschaftspotential in Multiple System Atrophy

**DOI:** 10.3389/fneur.2021.608322

**Published:** 2021-06-02

**Authors:** Yi-Chien Yang, Fang-Tzu Chang, Jui-Cheng Chen, Chon-Haw Tsai, Fu-Yu Lin, Ming-Kuei Lu

**Affiliations:** ^1^Department of Neurology, China Medical University Hospital, Taichung, Taiwan; ^2^School of Medicine, College of Medicine, China Medical University, Taichung, Taiwan; ^3^Department of Neurology, China Medical University Hsinchu Hospital, Hsinchu, Taiwan; ^4^Neuroscience and Brain Disease Center, China Medical University Hospital, Taichung, Taiwan; ^5^Ph.D. Program for Translational Medicine, College of Medicine, China Medical University, Taichung, Taiwan

**Keywords:** Bereitschaftspotential, movement-related cortical potential, multiple system atrophy, parkinsonism, neurodegenerative disorder

## Abstract

**Objective:** Multiple system atrophy (MSA) is a neurodegenerative disorder manifesting as parkinsonism, cerebellar ataxia, and autonomic dysfunction. It is categorized into MSA with predominant parkinsonism (MSA-P) and into MSA with predominant cerebellar ataxia (MSA-C). The pathophysiology of motor control circuitry involvement in MSA subtype is unclear. Bereitschaftspotential (BP) is a feasible clinical tool to measure electroencephalographic activity prior to volitional motions. We recorded BP in patients with MSA-P and MSA-C to investigate their motor cortical preparation and activation for volitional movement.

**Methods:** We included eight patients with MSA-P, eight patients with MSA-C, and eight age-matched healthy controls. BP was recorded during self-paced rapid wrist extension movements. The electroencephalographic epochs were time-locked to the electromyography onset of the voluntary wrist movements. The three groups were compared with respect to the mean amplitudes of early (1,500–500 ms before movement onset) and late (500–0 ms before movement onset) BP.

**Results:** Mean early BP amplitude was non-significantly different between the three groups. Mean late BP amplitude in the two patient groups was significantly reduced in the parietal area contralateral to the movement side compared with that in the healthy control group. In addition, the late BP of the MSA-C group but not the MSA-P group was significantly reduced at the central parietal area compared with that of the healthy control group.

**Conclusions:** Our findings suggest that patients with MSA exhibit motor cortical dysfunction in voluntary movement preparation and activation. The dysfunction can be practicably evaluated using late BP, which represents the cerebello-dentato-thalamo-cortical pathway.

## Introduction

Multiple system atrophy (MSA) is an adult-onset and progressive neurodegenerative disease. Unlike Parkinson's disease (PD), it has a disabling and rapidly fatal course. MSA is characterized by a combination of autonomic dysfunction, parkinsonism, cerebellar, and pyramidal features (1, 2). It is classified based on the motor phenotypes of striatonigral degeneration (SND), currently designated as MSA-parkinsonism type (MSA-P), and olivopontocerebellar atrophy (OPCA), currently designated as MSA-cerebellar type (MSA-C) (3). Despite the clinical manifestations show similar gender prevalence, age of onset and occurrence of orthostatic hypotension, the prognosis may be different between the two subtypes (4–6). Pathologically, MSA is a synucleinopathy in which the misfolded alpha-synuclein accumulates in the cytoplasmic inclusions of oligodendrocytes; this is different from PD, in which alpha-synuclein aggregates in the central nervous system neurons (7–9). Prominent degeneration and atrophy in the putamen, caudate nucleus, substantia nigra, pons, inferior olive, and cerebellum is observed in MSA compared with a predominant reduction in the caudate and putamen in patients with typical PD (1, 2, 10–12). Functional magnetic resonance imaging studies have revealed the disrupted functional connectivity of the cerebello-cortical pathway in patients with MSA and those with PD, but the patterns of the impaired connectivity differ between the two diseases (13–16). Despite sharing some parkinsonian features, MSA exhibits a distinct clinical course and symptom spectrum from PD (17, 18). Therefore, whether and how the underlying pathophysiology of MSA leads to motor dysfunction remains to be investigated.

Movement-related cortical potential (MRCP) represents the cortical neuronal activity corresponding to intentional movements (19–22). It is a useful tool in studying the motor initiation by evaluating both the cerebello-thalamo-cortical motor and cortico-basal ganglia-thalamo-cortical pathways (23–32). The Bereitschaftspotential (BP), defined as the pre-movement session of the MRCP, is particularly emphasized because it represents the preparatory and executive activation in the motor-related cortices. Early BP exhibits a slowly rising negative slope, usually arising from 1,500 to 500 milliseconds (ms) before movement onset, and late BP exhibits a steeper negative slope from 500 to 0 ms before the movement onset (22). Bilateral pre-supplementary motor area (pre-SMA) activation contributes to early BP, and the subsequent SMA proper, pre-motor and primary motor cortical activation contralateral to the movement contributes to late BP (22, 33).

The two BP components can be differently affected in patients with cerebellar disorders or basal ganglia lesions (23–28, 30, 31, 34, 35). In patients with cerebellar disorders, early BP is not affected, but late BP is reduced mostly over the central region of cortex, indicating dysfunction of the cerebello-dentato-thalamo-cortical pathway (23–28, 30, 31). In patients with PD, the early BP exhibits a smaller amplitude compared with healthy controls, probably due to the failure of SMA activation in the basal ganglia circuitry (34, 36). A study found reduced late BP in eight patients diagnosed with OPCA, according to the diagnosis consensus published in 1999 (37, 38). After the second diagnosis consensus of MSA in 2008 (39), no data are available to describe the full MRCP features, particularly BP, in the two subtypes of MSA. Although MSA-P and MSA-C share a common synucleinopathy at the cellular level, it is possible that different motor-related circuits are involved in these two subtypes of MSA. Here we adopted the new diagnostic criteria for MSA phenotypes and compared BP in patients with MSA-P, patients with MSA-C, and healthy controls.

In addition to BP, transcranial magnetic stimulation (TMS) is the other suitable non-invasive tool to investigate the function of the cerebello-thalamo-cortical circuit. In patients with PD, the cerebello-thalamo-cortical circuit has been found significantly impaired with a TMS technique targeting the cerebellar cortex and the contralateral primary motor cortex (40, 41). By this way the cerebellar modulatory influence on the corticospinal excitability can be measured. However, the cerebellar and the primary motor cortices are passively activated in the TMS studies. Since the current study focused on studying the volitional cortical activation in the two MSA subtypes, the TMS was not applied.

Clinical evidences have shown that different MSA subtypes would heterogeneously involve the cerebello-thalamo-cortical motor and the cortico-basal ganglia-thalamo-cortical pathways. BP might serve as a feasible tool to investigate the underlying dysfunction. Findings may elucidate motor control pathophysiology in the two MSA subtypes.

## Methods

### Patients

Sixteen patients with MSA (eight with MSA-P and eight with MSA-C) were included in this study (age: 62.1 ± 9.2 years; seven women and nine men) (Table 1). Eight age-matched patients without a history of neurological disorders were recruited as the control group (age: 60.9 ± 8.3 years; three women and five men). All patients were diagnosed with probable or possible MSA, according to the second consensus statement on the diagnosis of MSA (39), which was verified by two neurologists specializing in movement disorders. Patients with regular use of antiparkinsonian medications— including levodopa, catechol-O-methyltransferase inhibitors, dopamine agonists, anticholinergic agents, amantadine, and monoamine oxidase inhibitors—were requested to cease medication for at least 12 h before the examination. All participants were right-handed, according to the Edinburgh Handedness Inventory (43). This study was approved by the local ethics committee of the China Medical University Hospital (CMUH105-REC1-087). All participants provided their informed consent per the Declaration of Helsinki before participating in this study.

**Table 1:** Demographic data of patients with multiple system atrophy (MSA).

**Patient no**.	**Subtype**	**Age (years)/sex**	**Disease duration (years)**	**Levodopa equivalent dose (mg)**	**UMSARS part II**
1	MSA-P	70/M	7	450	36
2	MSA-P	71/M	4	250	26
3	MSA-P	78/M	10	1,040	26
4	MSA-P	68/M	5	800	10
5	MSA-P	64/M	4	500	29
6	MSA-P	59/F	7	315	19
7	MSA-P	66/F	5	1,170	33
8	MSA-P	80/M	2	200	17
9	MSA-C	56/M	1	300	13
10	MSA-C	54/F	2	300	27
11	MSA-C	57/F	2	525	29
12	MSA-C	54/M	1	400	16
13	MSA-C	49/F	15	150	20
14	MSA-C	56/F	10	900	37
15	MSA-C	55/M	4	500	31
16	MSA-C	57/F	1	200	21

*UMSARS, Unified Multiple System Atrophy Rating Scale (Part II score: 0–56) (42)*.

### Recording and Analysis

We used 26 Ag/AgCl scalp electroencephalogram (EEG) electrodes based on the international 10–20 EEG system. All electrodes were referenced to linked earlobe electrodes, and the signals were filtered with a bandpass ranging from 0.05 to 70 Hz (NeuroScan SynAmps, Neurosoft, Sterling, VA, USA). All electrodes had an impedance of <5 kΩ. Surface electromyogram (EMG) measurements were taken from the bilateral extensor carpi radialis (ECR) muscles. The surface EMG was rectified and filtered with a bandpass of 30–200 Hz. The EEG and surface EMG signals were simultaneously sampled, digitized at a rate of 1 kHz per channel and stored for offline analysis.

Participants were seated in a chair with their forearms and hands rested on the chair arms. We requested that they fix their gaze on a red spot 1.5 m ahead, and they performed self-paced, fast, and brisk volitional extensions of the unilateral wrist approximately every 5–7 s. All participants accomplished this task in 20 min for each hand.

**Figure 1:**
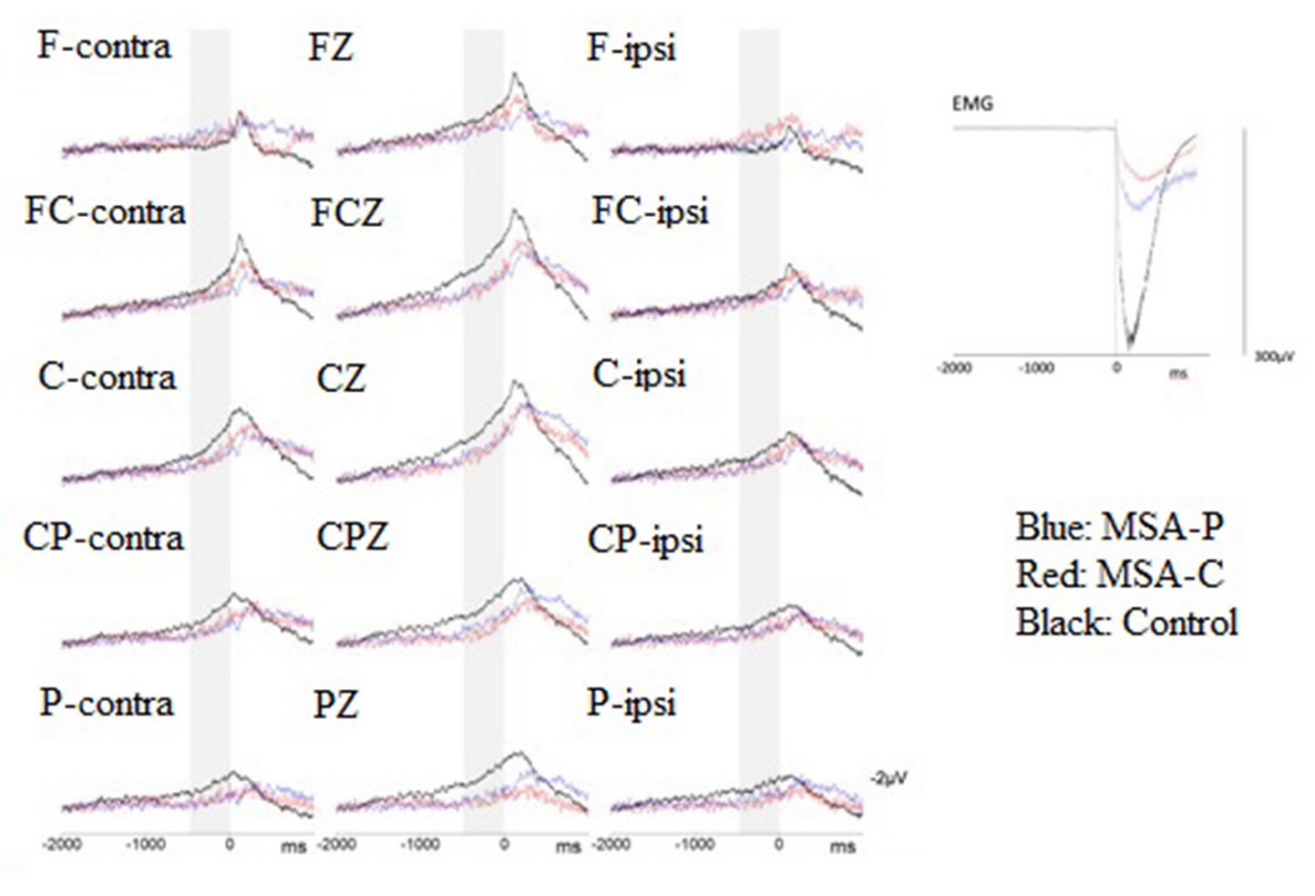
Superimposition of the grand-average Bereitschaftspotential (BP) from patients with multiple system atrophy (MSA)-P (*n* = 8, blue lines), patients with MSA-C (*n* = 8, red lines), and healthy controls (*n* = 8, black lines) were revealed at the 15 electrodes of interest when performing volition wrist extension. The zero denotes the onset of a surface electromyogram (sEMG) burst. Gray stripes represent the late BP period (500–0 ms before volitional sEMG burst onset). Grand averages of the voluntary sEMG burst during performance of wrist extension movements are shown at the right-hand part of this figure.

For BP analysis, we identified the EEG epoch from 2,000 ms before (−2,000 ms) to 1,000 ms after (+1,000 ms) the EMG burst onset. The mean amplitude of the initial 250 ms (−2,000 to −1,750 ms) was used as the baseline for each BP epoch. The onset of the surface EMG burst during each ECR muscle contraction was digitally marked as time zero after a visual inspection for every BP epoch. The EEG epochs that were contaminated by head muscle contraction or eyeball movement artifacts were excluded from further analysis. The artifact-free EEG sweeps were aligned with the rectified surface EMG burst onset and then averaged for the movement condition (i.e., right or left wrist extension movements) in each participant. The averaged BP waveforms were compared between the three groups (MSA-P, MSA-C, and control) (Figure 1). The BP from −1,500 to −500 ms in each epoch was defined as early BP and that from −500 to 0 ms was defined as late BP. We calculated the mean amplitude from −1,500 to −500 ms as the early BP amplitude, and the mean amplitude from −500 to 0 ms as the late BP amplitude. To compare the data obtained during the wrist movements of each hand, EEG data were replotted to indicate electrode positions contralateral (cont) or ipsilateral (ipsi) to the wrist movement. Data from 15 electrode locations (F-cont, Fz, F-ipsi, FC-cont, FCz, FC-ipsi, C-cont, Cz, C-ipsi, CP-cont, CPz, CP-ipsi, P-cont, Pz, and P-ipsi) were used for statistical analysis.

### Statistical Analysis

We used SPSS for Windows Version 22.0. For BP amplitude analysis, repeated-measures analysis of variance (ANOVA) was performed. The within-subject factors were location (the 15 electrode locations) and movement side (right and left). The between-subject factor was the group (MSA-P, MSA-C, and control). To determine significance in the *F*-value, *post-hoc* between-group comparisons were performed using a one-way ANOVA test with Fisher's least significant difference. For comparisons of the surface EMG onset-to-peak time and the peak amplitude, the Kruskal–Wallis-test was performed. The Mann–Whitney-test was used for *post-hoc* analysis of between-group comparisons. A *p*-value < 0.05 indicated statistical significance.

## Results

### Bereitschaftspotential Analysis

**Figure 2:**
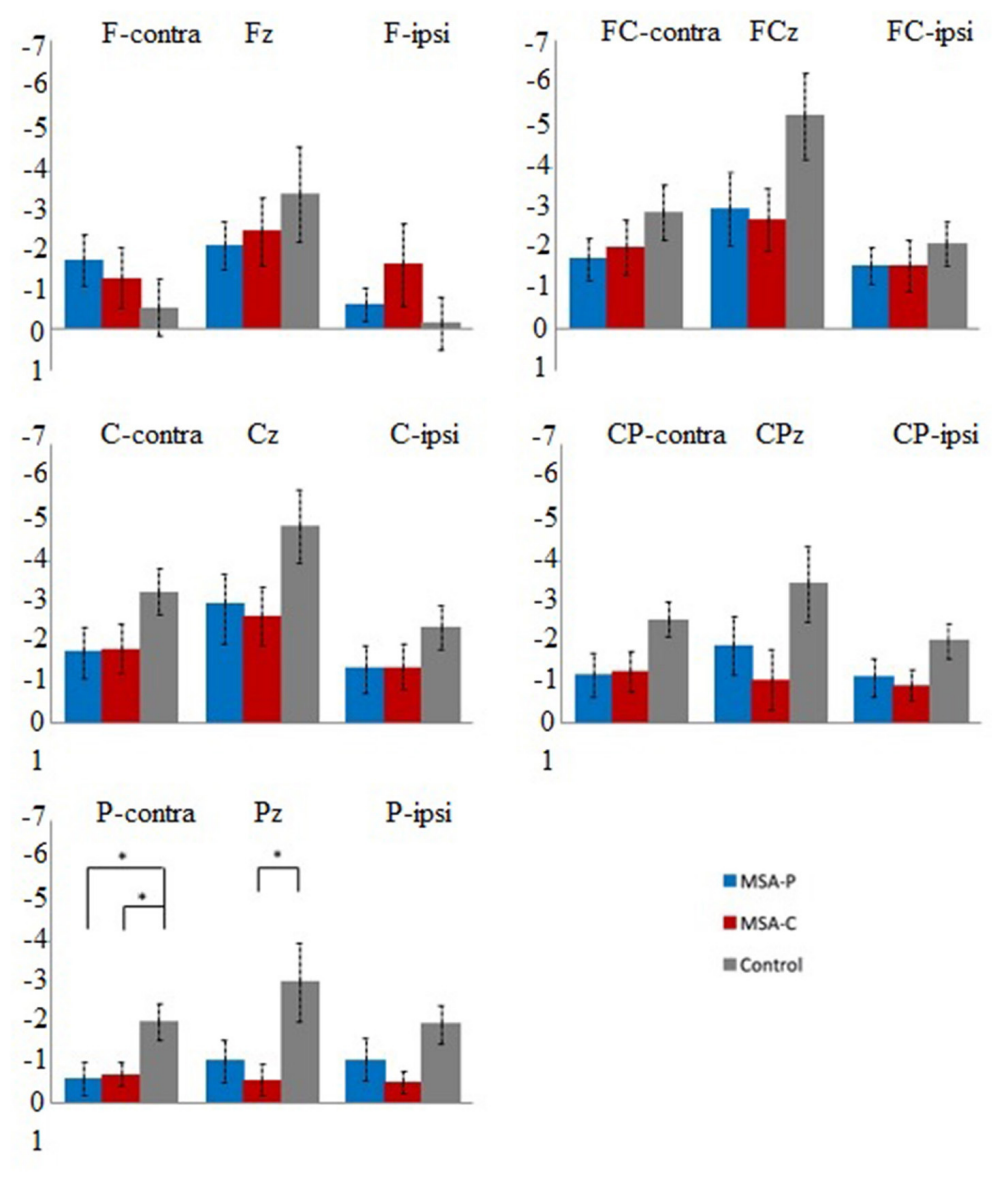
*Post-hoc* comparisons of the mean late Bereitschaftspotential (BP) amplitude (μV) between patients with multiple system atrophy (MSA)-P (blue), patients with MSA-C (red), and healthy controls (black) at the 15 electrodes of interest. Error bars indicate standard errors. *p < 0.05.

Since the movement side did not show any significant interactions with location and/or group on early and late BP in the repeated-measures ANOVA (all *p* > 0.08), we rearranged all electrodes to either the ipsilateral or contralateral group corresponding to the movement side (31). The repeated-measures ANOVA revealed a significant effect for the interaction of location and group on late BP (*F* = 2.940, *p* = 0.006; Table 2). This effect was not significant on early BP (*F* = 1.972, *p* = 0.068; Table 2). For late BP, we further explored the interaction of location and group in the *post-hoc* comparisons for MSA-P, MSA-C, and the control (Figure 2). The amplitudes of late BP exhibited a significant difference in P-cont and Pz between MSA-P, MSA-C, and control groups (*p* < 0.05). On the electrode P-cont, *post-hoc* comparisons revealed a significant decrease in the mean amplitude of late BP in the MSA-P group compared with that in the control group (−0.62 ± 1.19 vs. −2.0 ± 1.26 μV, *p* < 0.05). The mean amplitude of late BP was also significantly reduced in the MSA-C group compared with that in the control group (−0.70 ± 0.83 vs. −2.0 ± 1.26 μV, *p* < 0.05). On the electrode PZ, *post-hoc* comparisons revealed a significant decrease of the mean amplitude of late BP in the MSA-C group compared with that of the control group (−0.58 ± 1.13 vs. −2.99 ± 2.80 μV, *p* < 0.05).

**Table 2:** Repeated-measures analysis of variance of mean amplitudes of early and late Bereitschaftspotential (BP).

	**Early BP**		**Late BP**	
	***F***	***P***	***F***	***P***
**Within subject factor**				
Location^a^	10.442	<0.001^*^	16.386	<0.001^*^
**Between subject factor**				
Group^b^	2.231	0.132	1.262	0.304
Location^a^ × group^b^	1.972	0.068	2.940	0.006^*^

*Early BP: 1,500–500 ms before movement onset; late BP: 500–0 ms before movement onset*.

a *15 levels, including F-cont, Fz, F-ipsi, FC-cont, FCz, FC-ipsi, C-cont, Cz, C-ipsi, CP-cont, CPz, CP-ipsi, P-cont, Pz, and P-ipsi*.

b *3 levels, including patients with MSA-P, patients with MSA-C, and healthy controls*.

*Bold means*

* *p < 0.05*.

### Movement Performance

The number of the movements acquired from the right wrist movement was 117, 123, and 210 in the patients with MSA-P, MSA-C and healthy subjects, respectively. There were 109, 116, and 213 movements acquired from the left wrist movement in the same three groups. The number of the artifact-free EEG epochs was 69, 68, and 115 on right wrist movement in the patients with MSA-P, MSA-C and healthy subjects, respectively. On left wrist movement, the artifact-free EEG epochs were 66, 72, and 119 in the same three groups.

Movement performance was measured using surface EMG signals during wrist extension. We analyzed the onset-to-peak time and the peak amplitude of the single-trial rectified and average surface EMG. For onset-to-peak time, a significant difference was noted between three groups (MSA-P, MSA-C, and control: 300.8 ± 102.0 ms, 395.5 ± 66.4 ms, and 186.8 ± 90.2 ms, respectively). In the *post-hoc* analyses, the difference was significant between the MSA-C and control groups (*p* < 0.05) and the MSA-P and control groups (*p* < 0.05) but not between the MSA-P and MSA-C groups. For the peak amplitude, a significant difference was observed between the three groups (MSA-P, MSA-C, and control: 164.9 ± 153.0 μV, 96.2 ± 65.2 V, and 385.8 ± 214.5 μV, respectively). In the *post-hoc* analyses, the difference was significant between the MSA-C and control groups (*p* < 0.05) but not between the MSA-P and control groups or the MSA-P and MSA-C groups.

## Discussion

Our data suggested that MRCP in patients with MSA-P and MSA-C was different than that in the healthy controls. In brief, the late BP amplitude was significantly reduced on the contralateral parietal area (i.e., the P-cont electrode) in both MSA phenotypes. The MSA-C group exhibited a more extensive reduction of the late BP amplitude (i.e., involving Pz and P-cont electrodes) than the MSA-P group (i.e., involving only the P-cont electrode). The findings are consistent with a previous study demonstrating that late BP was smaller in patients with OPCA compared with the controls (37). The differences in early BP between MSA-P, MSA-C, and control groups were not robust. Because patients with PD had a profound early BP reduction in the frontocentral area (i.e., FCZ and CZ) (22, 34), our findings imply that different phases of volitional movement preparation are affected in PD and MSA.

### Distinct Motor Networks Between PD and MSA

Early BP in patients with PD is smaller than that in healthy individuals over frontocentral brain regions (34). Early BP may be generated from the bilateral pre-SMA and pre-motor areas with a midline maximal and symmetrical distribution (22). Thus, the reduction of the early BP in patients with PD may be due to insufficient afferents from the basal ganglia to the SMA region *via* the basal ganglia-thalamo-cortical pathway, indicating the involvement of the frontal basal ganglia circuitry in the motor impairment of PD (34, 44, 45). The manifestation of the late BP in PD, however, has been inconsistent, ranging from normal to larger-than-normal amplitudes (33, 34, 46). Compared with the control group, patients with PD may exhibit greater late BP, which suggests compensatory activation from the other brain regions, probably the cerebellum (33, 34).

In our study, patients with MSA-P or MSA-C exhibited no difference in early BP compared with healthy controls, but the reduction in late BP was significant at the contralateral parietal region. This discrepancy may account for the pathophysiological differences between MSA and PD. The cerebellar involvement suggested a distinct mechanism between MSA and PD. In a functional magnetic resonance imaging (fMRI) study, patients with MSA exhibited a more widespread reduction of brain activities than patients with PD did in the primary motor cortex, SMA, and superior cerebellum (47). In a positron emission tomography (PET) study, additional cerebellar activation was observed in patients with PD, potentially compensating for the cortico-basal ganglia dysfunction (48, 49). Frontoparietal cortical activation, rather than cerebellar activation, was revealed in patients with MSA, indicating cerebellar dysfunction in MSA (50–52). The aforementioned studies have indicated the role of cerebellar impairment in MSA motor control circuitry. Our finding of a reduction in the late BP amplitude in patients with MSA-P and MSA-C constitutes electrophysiological evidence for cerebellar involvement.

### Reduction of the Late BP in the Contralateral Parietal Region in MSA

In our study, patients with MSA-P and MSA-C exhibited a notable reduction of the late BP amplitude mainly in the contralateral parietal region compared with the healthy controls. The late BP has been postulated to begin in the lateral pre-motor and primary motor cortex, mainly on the contralateral side, receiving cerebellar projections from the contralateral dentate nucleus with a relay on the thalamus, namely the cerebello-dentato-thalamo-cortical pathway. The parietal lobe affects motor control, prediction, and coordination with the cerebellum (53–55). The cerebellum projects to the parietal region for the adaptive control of visuo-motor guidance in reaching and coordination (56, 57).

Changes in the late BP have been also reported in various cerebellar lesions (23, 24, 26, 28). In patients with dentate nucleus lesions, the late BP amplitudes were absent or markedly reduced, indicating that the dentate nucleus may play a role on the generation of the late BP (28). In patients with spinocerebellar ataxia type 3, the early BP was not affected, but the decreased late BP amplitudes in the central area and in the region contralateral to the movement site were noted (30). Patients with advanced essential tremor having intension tremor, a sign of cerebellar dysfunction, exhibited a significant reduction in the late BP (31). In both these patient groups, the reduced late BP amplitudes may be attributed to an insufficient excitatory efferent from the dentate nuclei to the motor cortex (30, 31).

In patients with MSA, brain single-photon emission tomography imaging revealed a markedly reduced perfusion in the striatum, brain stem, and cerebellum compared with patients with PD (58). A task-based fMRI study indicated a more widespread reduction in functional activity in the primary motor cortex, SMA, and superior cerebellum in MSA compared with PD (47). In our study, a decreased late BP amplitude at contralateral parietal lobes in both MSA-P and MSA-C groups suggests that dysfunction of the cerebello-dentato-thalamo-cortical pathway with widespread cortical involvement, such as that of the parietal lobe, contributes to the loss of motor control in MSA.

### Pathophysiology Between MSA-P and MSA-C

Currently, the classification of MSA-P and MSA-C are diagnosed on the basis of clinical presentation (39), and there exist a paucity of reliable biomarkers and imaging tools to differentiate them. The pathophysiological differences between them has also drawn much attention. In a PET study, MSA-P and MSA-C exhibited different patterns of dopamine transporter loss in striatal regions (59, 60). Our findings demonstrated a more extensive cortical involvement in MSA-C (i.e., P-cont and Pz) than in MSA-P (i.e., P-cont). Although no significant difference was observed between MSA-P and MSA-C, which was probably due to the small number of cases and lack of statistical power of our study, the difference in BP changes between MSA-P and MSA-C should be further investigated in future studies.

### Study Limitations

First, movement performance data from EMG analysis indicated significantly prolonged latencies and reduced amplitudes from EMG onset to EMG peak in both MSA-P and MSA-C groups, compared with healthy controls; however, this was inevitable owing to bradykinesia and bradyphrenia in the patients with MSA-P and MSA-C. Second, our study did not recruit patients with PD; therefore, no directly comparable data were available between the PD, MSA-P, and MSA-C groups. Third, the sample size in our study was small. The possibility of statistical underestimation due to a type II error cannot be excluded. Larger sample sizes are warranted to verify our findings. Additionally, a complementary tool such as TMS would be also helpful to consolidate our hypothesis.

## Conclusion

This study provided evidence showing abnormal BP in patients with MSA. The decreased late BP amplitude in patients with MSA-P and MSA-C indicated a specific motor cortical dysfunction corresponding to voluntary movement preparation and initiation. The functional role of the cerebello-dentato-thalamo-cortical pathway, which mediates late BP in MSA requires further study.

## Data Availability Statement

The raw data supporting the conclusions of this article will be made available by the authors, without undue reservation.

## Ethics Statement

The studies involving human participants were reviewed and approved by the Ethics Committee of China Medical University Hospital. The patients/participants provided their written informed consent to participate in this study.

## Author Contributions

Y-CY: conception of the work, data acquisition, statistical analysis, and draft writing. F-TC: conception of the work and draft writing. J-CC: clinical evaluation and long-term follow-up of patients. C-HT: conception and organization of the work, clinical evaluation, and long-term follow-up of patients. F-YL: conception of the work, data acquisition, statistical analysis, and manuscript review. M-KL: conception of the work, statistical analysis, clinical evaluation, long-term follow-up of patients, and manuscript revision. All authors contributed to the article and approved the submitted version.

## Conflict of Interest

The authors declare that the research was conducted in the absence of any commercial or financial relationships that could be construed as a potential conflict of interest.

## References

[1] Fanciulli A Wenning GK. Multiple-system atrophy. N Engl J Med. (2015) 372:249–63. 10.1056/NEJMra1311488 25587949

[2] Krismer F Wenning GK. Multiple system atrophy: insights into a rare and debilitating movement disorder. Nat Rev Neurol. (2017) 13:232–43. 10.1038/nrneurol.2017.26 28303913

[3] Laurens B Vergnet S Lopez MC Foubert-Samier A Tison F Fernagut PO. Multiple system atrophy—state of the art. Curr Neurol Neurosci Rep. (2017) 17:41. 10.1007/s11910-017-0751-0 28378233

[4] Watanabe H Saito Y Terao S Ando T Kachi T Mukai E. Progression and prognosis in multiple system atrophy: an analysis of 230 Japanese patients. Brain. (2002) 125:1070–83. 10.1093/brain/awf117 11960896

[5] Ozawa T Tada M Kakita A Onodera O Tada M Ishihara T. The phenotype spectrum of Japanese multiple system atrophy. J Neurol Neurosurg Psychiatry. (2010) 81:1253e1255. 10.1136/jnnp.2009.182576 20571046

[6] Roncevic D Palma J-A Martinez J Goulding N Norcliffe-Kaufmann L. Kaufmann H. Cerebellar and parkinsonian phenotypes in multiple system atrophy: similarities, differences and survival. J Neural Transm. (2014) 121:507–12. 10.1007/s00702-013-1133-7 24337696 PMC4134009

[7] McCann H Stevens CH Cartwright H Halliday MG. α-Synucleinopathy phenotypes. Parkinsonism Relat Disord. (2014) 20(Suppl. 1):S62–7. 10.1016/S1353-8020(13)70017-8 24262191

[8] Jellinger KA. Multiple system atrophy: an oligodendroglioneural synucleinopathy. J Alzheimers Dis. (2018) 62:1141–79. 10.3233/JAD-170397 28984582 PMC5870010

[9] Valdinocci D Radford RAW Goulding M Hayashi J Chung RS Pountney LD. Extracellular interactions of alpha-synuclein in multiple system atrophy. Int J Mol Sci. (2018) 19:4129. 10.3390/ijms19124129 30572656 PMC6320782

[10] Pitcher TL Melzer TR Macaskill MR Graham CF Livingston L Keenan RJ. Reduced striatal volumes in Parkinson's disease: a magnetic resonance imaging study. Transl Neurodegener. (2012) 1:17. 10.1186/2047-9158-1-17 23210661 PMC3514123

[11] Fanciulli A Stankovic I Krismer F Seppi K Levin J Wenning KG. Multiple system atrophy. Int Rev Neurobiol. (2019) 149:137–92. 10.1016/bs.irn.2019.10.004 31779811

[12] Wang N Zhang L Yang H Liu H Luo X Fan G. Similarities and differences in cerebellar grey matter volume and disrupted functional connectivity in idiopathic Parkinson's disease and multiple system atrophy. Neuropsychologia. (2019) 124:125–32. 10.1016/j.neuropsychologia.2018.12.019 30590063

[13] Wang N Edmiston EK Luo X Yang H Chang M Wang F. Comparing abnormalities of amplitude of low-frequency fluctuations in multiple system atrophy and idiopathic Parkinson's disease measured with resting-state fMRI. Psychiatry Res Neuroimaging. (2017) 269:73–81. 10.1016/j.pscychresns.2017.09.002 28957750

[14] Yao Q Zhu D Li F Xiao C Lin X Huang Q. Altered functional and causal connectivity of cerebello-cortical circuits between multiple system atrophy (parkinsonian type) and Parkinson's Disease. Front Aging Neurosci. (2017) 9:266–6. 10.3389/fnagi.2017.00266 28848423 PMC5554370

[15] Ren S Zhang H Zheng W Liu M Gao F Wang Z. Altered functional connectivity of cerebello-cortical circuit in multiple system atrophy (cerebellar-type). Front Neurosci. (2018) 12:996. 10.3389/fnins.2018.00996 30662394 PMC6328464

[16] Baggio HC Abos A Segura B Campabadal A Uribe C Giraldo DM. Cerebellar resting-state functional connectivity in Parkinson's disease and multiple system atrophy: characterization of abnormalities and potential for differential diagnosis at the single-patient level. Neuroimage Clin. (2019) 22:101720. 10.1016/j.nicl.2019.10172 30785051 PMC6383182

[17] Keener AM Bordelon YM. Parkinsonism. Semin Neurol. (2016) 36:330–4. 10.1055/s-0036-1585097 27643900

[18] McFarland NR. Diagnostic approach to atypical parkinsonian syndromes. Continuum (Minneap Minn). (2016) 22:1117–42. 10.1212/CON.0000000000000348 27495201 PMC5567217

[19] Shibasaki H Barrett G Halliday E Halliday MA. Components of the movement-related cortical potential and their scalp topography. Electroencephalogr Clin Neurophysiol. (1980) 49:213–26. 10.1016/0013-4694(80)90216-3 6158398

[20] Deecke L. Electrophysiological correlates of movement initiation. Rev Neurol (Paris). (1990) 146:612–9. 2263824

[21] Deecke L. The Bereitschaftspotential as an electrophysiological tool for studying the cortical organization of human voluntary action. Suppl Clin Neurophysiol. (2000) 53:199–206. 10.1016/s1567-424x(09)70158-8 12740997

[22] Shibasaki H Hallett M. What is the bereitschaftspotential? Clin Neurophysiol. (2006) 117:2341–56. 10.1016/j.clinph.2006.04.025 16876476

[23] Shibasaki H Shima F Kuroiwa Y. Clinical studies of the movement-related cortical potential (MP) and the relationship between the dentatorubrothalamic pathway and readiness potential (RP). J Neurol. (1978) 219:15–25. 10.1007/BF00313365 81281

[24] Shibasaki H Barrett G Neshige R Hirata I Tomoda H. Volitional movement is not preceded by cortical slow negativity in cerebellar dentate lesion in man. Brain Res. (1986) 368:361–5. 10.1016/0006-8993(86)90582-2 3697731

[25] Fève A Bathien N Rondot P. Abnormal movement related potentials in patients with lesions of basal ganglia and anterior thalamus. J Neurol Neurosurg Psychiatry. (1994) 57:100–4. 10.1136/jnnp.57.1.100 8301287 PMC485047

[26] Ikeda A Shibasaki H Nagamine T Terada K Kaji R Fukuyama H. Dissociation between contingent negative variation and bereitschaftspotential in a patient with cerebellar efferent lesion. Electroencephalogr Clin Neurophysiol. (1994) 90:359–64. 10.1016/0013-4694(94)90051-5 7514982

[27] Wessel K Verleger R Nazarenus D Vieregge P Kömpf D. Movement-related cortical potentials preceding sequential and goal-directed finger and arm movements in patients with cerebellar atrophy. Electroencephalogr Clin Neurophysiol. (1994) 92:331–41. 10.1016/0168-5597(94)90101-5 7517855

[28] Kitamura J Shabasaki H Terashi A Tashima K. Cortical potentials preceding voluntary finger movement in patients with focal cerebellar lesion. Clin Neurophysiol. (1999) 110:126–32. 10.1016/s0168-5597(98)00052-5 10348331

[29] Rektor I Bares M Kubová D. Movement-related potentials in the basal ganglia: a SEEG readiness potential study. Clin Neurophysiol. (2001) 112:2146–53. 10.1016/s1388-2457(01)00662-9 11682354

[30] Lu MK Shih HT Huang KJ Ziemann U Tsai CH Chang FC. Movement-related cortical potentials in patients with Machado-Joseph disease. Clin Neurophysiol. (2008) 119:1010–9. 10.1016/j.clinph.2008.01.008 18334306

[31] Lu MK Jung P Bliem B Shih HT Hseu YT Yang YW. The Bereitschaftspotential in essential tremor. Clin Neurophysiol. (2010) 121:622–30. 10.1016/j.clinph.2009.12.014 20097128

[32] Milardi D Quartarone A Bramanti A Anastasi G Bertino S Basile GA. The cortico-basal ganglia-cerebellar network: past, present and future perspectives. Front Syst Neurosci. (2019) 13:61. 10.3389/fnsys.2019.00061 31736719 PMC6831548

[33] Colebatch JG. Bereitschaftspotential and movement-related potentials: origin, significance, and application in disorders of human movement. Mov Disord. (2007) 22:601–10. 10.1002/mds.21323 17260337

[34] Dick JP Rothwell JC Day BL Cantello R Buruma O Gioux M. The bereitschaftspotential is abnormal in Parkinson's disease. Brain. (1989) 112:233–44. 10.1093/brain/112.1.233 2917279

[35] Georgiev D Lange F Seer C Kopp B Jahanshahi M. Movement-related potentials in Parkinson's disease. Clin Neurophysiol. (2016) 127:2509–19. 10.1016/j.clinph.2016.04.004 27178872

[36] Galvan A Devergnas A Wichmann T. Alterations in neuronal activity in basal ganglia-thalamocortical circuits in the parkinsonian state. Front Neuroanat. (2015) 9:5. 10.3389/fnana.2015.00005 25698937 PMC4318426

[37] Oishi M Mochizuki Y Takasu T. Movement-related cortical potentials and contingent negative variation in olivopontocerebellar atrophy. Clin Electroencephalogr. (1997) 28:245–8. 10.1177/155005949702800410 9343719

[38] Gilman S Low PA Quinn N Albanese A Ben-Shlomo Y Fowler CJ. Consensus statement on the diagnosis of multiple system atrophy. J Neurol Sci. (1999) 163:94–8. 10.1016/s0022-510x(98)00304-9 10223419

[39] Gilman S Wenning GK Low PA Brooks DJ Mathias CJ Trojanowski JQ. Second consensus statement on the diagnosis of multiple system atrophy. Neurology. (2008) 71:670–6. 10.1212/01.wnl.0000324625.00404.15 18725592 PMC2676993

[40] Schirinzi T Lorenzo FD Ponzo V Palmieri MG Bentivoglio AR Schillaci O. Mild cerebello-thalamo-cortical impairment in patients with normal dopaminergic scans (SWEDD). Parkinsonism Relat Disord. (2016) 28:23–8. 10.1016/j.parkreldis.2016.03.023 27170027

[41] Carrillo F Palomar FJ Conde V Diaz-Corrales FJ Porcacchia P Fernández-del-Olmo M. Study of cerebello-thalamocortical pathway by transcranial magnetic stimulation in Parkinson's disease. Brain Stimul. (2013) 6:582–9. 10.1016/j.brs.2012.12.004 23318222

[42] Wenning GK Tison F Seppi K Sampaio C Diem A Yekhlef F. Development and validation of the unified multiple system atrophy rating scale (UMSARS). Mov Disord. (2004) 19:1391–402. 10.1002/mds.20255 15452868

[43] Oldfield RC. The assessment and analysis of handedness: the Edinburgh inventory. Neuropsychologia. (1971) 9:97–113. 5146491 10.1016/0028-3932(71)90067-4

[44] Gerlach M Gsell W Kornhuber J Jellinger K Krieger V Pantucek F. A post mortem study on neurochemical markers of dopaminergic, GABA-ergic and glutamatergic neurons in basal ganglia-thalamocortical circuits in Parkinson syndrome. Brain Res. (1996) 741:142–52. 10.1016/s0006-8993(96)00915-8 9001716

[45] Wu T Wang J Wang C Hallett M Zang Y Wu X. Basal ganglia circuits changes in Parkinson's disease patients. Neurosci Lett. (2012) 524:55–9. 10.1016/j.neulet.2012.07.012 22813979 PMC4163196

[46] Jahanshahi M Jenkins IH Brown RG Marsden CD Passingham RE Brooks JD. Self-initiated versus externally triggered movements. I. An investigation using measurement of regional cerebral blood flow with PET and movement-related potentials in normal and Parkinson's disease subjects. Brain. (1995) 118:913–33. 10.1093/brain/118.4.913 7655888

[47] Burciu RG Chung JW Shukla P Ofori E Li H McFarland NR. Functional MRI of disease progression in Parkinson disease and atypical parkinsonian syndromes. Neurology. (2016) 87:709–17. 10.1212/WNL.0000000000002985 27421545 PMC4999161

[48] Thobois S Jahanshahi M Pinto S Frackowiak R Limousin-Dowsey P. PET and SPECT functional imaging studies in Parkinsonian syndromes: from the lesion to its consequences. Neuroimage. (2004) 23:1–16. 10.1016/j.neuroimage.2004.04.039 15325346

[49] Wu T Hallett M. The cerebellum in Parkinson's disease. Brain. (2013) 136:696–709. 10.1093/brain/aws360 23404337 PMC7273201

[50] Payoux P Brefel-Courbon C Ory-Magne F Regragui W Thalamas C Balduyck S. Motor activation in multiple system atrophy and Parkinson disease: a PET study. Neurology. (2010) 75:1174–80. 10.1212/WNL.0b013e3181f4d78f 20876470

[51] Lu CF Soong BW Wu HM Teng S Wang PS Wu TY. Disrupted cerebellar connectivity reduces whole-brain network efficiency in multiple system atrophy. Mov Disord. (2013) 28:362–9. 10.1002/mds.25314 23325625

[52] Wang H Li L Wu T Hou B Wu S Qiu Y. Increased cerebellar activation after repetitive transcranial magnetic stimulation over the primary motor cortex in patients with multiple system atrophy. Ann Transl Med. (2016) 4:103. 10.21037/atm.2016.03.24 27127756 PMC4828746

[53] Blakemore SJ Sirigu A. Action prediction in the cerebellum and in the parietal lobe. Exp Brain Res. (2003) 153:239–45. 10.1007/s00221-003-1597-z 12955381

[54] Pelton TA Wing AM Fraser D van Vliet P. Differential effects of parietal and cerebellar stroke in response to object location perturbation. Front Hum Neurosci. (2015) 9:293. 10.3389/fnhum.2015.00293 26217208 PMC4499699

[55] Ramnani N Toni I Passingham RE Haggard P. The cerebellum and parietal cortex play a specific role in coordination: a PET study. Neuroimage. (2001) 14:899–911. 10.1006/nimg.2001.0885 11554809

[56] Allen G McColl R Barnard H Ringe WK Fleckenstein J Cullum CM. Magnetic resonance imaging of cerebellar-prefrontal and cerebellar-parietal functional connectivity. Neuroimage. (2005) 28:39–48. 10.1016/j.neuroimage.2005.06.013 16023375

[57] Prevosto V Graf W Ugolini G. Cerebellar inputs to intraparietal cortex areas LIP and MIP: functional frameworks for adaptive control of eye movements, reaching, and arm/eye/head movement coordination. Cereb Cortex. (2010) 20:214–28. 10.1093/cercor/bhp091 19465740 PMC2860711

[58] Cilia R Marotta G Benti R Pezzoli G Antonini A. Brain SPECT imaging in multiple system atrophy. J Neural Transm (Vienna). (2005) 112:1635–45. 10.1007/s00702-005-0382-5 16284908

[59] Kim HW Kim JS Oh M Oh JS Lee SJ Oh SJ. Different loss of dopamine transporter according to subtype of multiple system atrophy. Eur J Nucl Med Mol Imaging. (2016) 43:517–25. 10.1007/s00259-015-3191-6 26384682

[60] Bu LL Liu FT Jiang CF Guo SS Yu H Zuo CT. Patterns of dopamine transporter imaging in subtypes of multiple system atrophy. Acta Neurol Scand. (2018) 138:170–6. 10.1111/ane.12932 29573392

